# RE: The effectiveness of telepsychiatry: thematic review

**DOI:** 10.1192/bjb.2023.82

**Published:** 2023-12

**Authors:** Imani Nwokeji, Oluwafisayo Adeoye, Chisom Madu

**Affiliations:** Medical Student, City University of New York School of Medicine, New York, NY, USA. Email: inwokej000@citymail.cuny.edu; Medical Student, City University of New York School of Medicine, New York, NY, USA. Email: oadeoye000@citymail.cuny.edu; Medical Student, City University of New York School of Medicine, New York, NY, USA. Email: cmadu000@citymail.cuny.edu

## The call for virtual elective clinical rotations for future psychiatrists

In recent years, telepsychiatry has proven crucial in access to mental healthcare, especially with the increased usage of virtual platforms to practise medicine. The virtual option of psychiatry has expanded substantially, providing care to underserved populations and addressing gaps in access to care in adverse populations.^1^ In their thematic review describing telepsychiatry as a promising method of practising psychiatry, Sharma et al identified five themes that are associated with the effectiveness of telepsychiatry: patient and clinician satisfaction, technology, diagnostic reliability, outcomes and professional guidance. However, the main barriers that limit the number of psychiatrists that participate in telepsychiatry include reluctance among clinicians and lack of professional guidance. The authors recommend education on the uses of telepsychiatry among clinicians and the provision of professional guidance for future practice. Telepsychiatry is concluded to be a cost-effective way to enhance mental healthcare that is beneficial to the patients of disadvantaged populations. This specifically benefits patients with limited mobility, those living in rural areas and patients that are incarcerated.^1,2^

Given the benefits of telepsychiatry to underserved communities, there is a need to promote its use. Strategies to provide more equitable mental health services and have more clinicians inclined to participate in telepsychiatry begin with the education of future clinicians. Previous studies have indicated the positive implications of residents participating in on-calls virtually. Particularly, residents felt more confident and equipped to treat patients traditionally and virtually. They made fewer medical errors and reported lower levels of burnout, stress and anxiety.^2^ In addition, residents that participated in virtual on-call programmes reported feeling more supported by their attending physicians as well as by other healthcare providers.^2,3^

With the goal of improving the experience of telepsychiatry for both patients and clinicians, an option for virtual elective clinical rotations during medical school training could be implemented for medical students across the USA. For students interested in psychiatry, this could prove to be extremely impactful. Future clinicians would be more equipped to deal with the virtual changes that have drastically altered the accessibility of care for patients.^3^ Furthermore, previous literature suggests many benefits of virtual electives, including increasing access to educational opportunities, reducing costs, increasing flexibility and enhancing learning outcomes.^4^

In summary, telepsychiatry is a rapidly evolving field, and current clinicians are struggling to progress with the ever-growing implementations of virtual calls and the positive effects for psychiatry residents. The option of virtual elective clinical rotations would allow medical students more viability to fully experience the realm of psychiatry today and become more equipped to practise telepsychiatry.^4^ This is significant, as previous studies have indicated that residents participating in such rotations had fewer errors during their training and were able to provide care to communities with limited access.^5^ Ultimately, this cost-effective manner of treating patients has proved to be extremely beneficial. For future physicians, virtual elective clinical rotations should be offered to medical students, especially those interested in practising psychiatry.
